# Poly(butylene 2,5-thiophenedicarboxylate): An Added Value to the Class of High Gas Barrier Biopolyesters

**DOI:** 10.3390/polym10020167

**Published:** 2018-02-09

**Authors:** Giulia Guidotti, Matteo Gigli, Michelina Soccio, Nadia Lotti, Massimo Gazzano, Valentina Siracusa, Andrea Munari

**Affiliations:** 1 Department of Civil, Chemical, Environmental and Materials Engineering, University of Bologna, Via Terracini 28, 40131 Bologna, Italy; giulia.guidotti9@unibo.it (G.G.); m.soccio@unibo.it (M.S.); andrea.munari@unibo.it (A.M.); 2 Department of Chemical Science and Technologies, University of Rome Tor Vergata, Via della Ricerca Scientifica 1, 00133 Roma, Italy; 3 Organic Synthesis and Photoreactivity Institute, CNR, Via Gobetti 101, 40129 Bologna, Italy; massimo.gazzano@cnr.it; 4 Department of Chemical Science, University of Catania, Viale A. Doria 6, 95125 Catania, Italy; vsiracus@dmfci.unict.it

**Keywords:** poly(butylene 2,5-thiophenedicarboxylate), biopolymers, *meso*-phase, mechanical properties, gas barrier properties

## Abstract

Many efforts are currently devoted to the design and development of high performance bioplastics to replace traditional fossil-based polymers. In response, this contribution presents a new biobased aromatic polyester, i.e., poly(butylene 2,5-thiophenedicarboxylate) (PBTF). Here, PBTF is characterized from the molecular, thermo-mechanical and structural point of view. Gas permeability is evaluated at different temperatures, in the range below and above glass transition, providing a full insight into the performances of this material under different operating conditions, and demonstrating the superior gas barrier behavior of PBTF with respect to other polyesters, such as PEF and PET. The combination of calorimetric and diffractometric studies allows for a deep understanding of the structure of PBTF, revealing the presence of a not-induced 2D-ordered phase (*meso*-phase), responsible for its outstanding gas permeability behavior. The simple synthetic strategy adopted, the exceptional barrier properties, combined with the interesting mechanical characteristics of PBTF open up new scenarios in the world of green and sustainable packaging materials.

## 1. Introduction

In recent years, great attention has been paid to the exploitation of renewable resources in response to fossil fuel depletion and growing environmental pollution. Plastic materials, which nowadays are employed in nearly all aspects of our daily life, are not an exception.

Indeed, global plastic production has constantly increased, reaching 322 Mtons in 2015 [1]. This generates huge amounts of plastic trash (about 30 Mtons/year only in Europe) that end up continuously in the waste stream, causing well-known terrestrial and aquatic contamination problems. In this respect, packaging probably represents the most pressing issue for various reasons: it accounts for ca. 40% of global plastic demand [1], it is a disposable item and, in case of food contact, its recycling is not very desirable [2].

Therefore, the use of bioplastics, i.e., plastics obtained from renewable resources and/or biodegradable, may represent a solution to these urgent needs. As reported by the European Bioplastics, their actual market is 4.1 Mtons/year and the growth expectations are about 50% in 5 years, thus at a much higher rate with respect to traditional plastics [3].

Many natural polymers such as starch, chitin, lignin, cellulose and vegetable oils have been extensively studied to promote the shift toward a greener economy. More recently, the scientific community has devoted its efforts to the synthesis of bio-based monomers for the production of highly performing biopolymers. In this framework, aliphatic polyesters (APs) are probably the most successful example, since various products based on poly(lactic acid), polyhydroxyalcanoates, poly(alkylene succinate)s and poly(alkylene adipate)s have been brought to the market. Although all commercially available, none of the above-mentioned APs displays gas barrier properties high enough to be considered a real alternative to poly(ethylene terephthalate) (PET). In the last years, another class of biopolymers received considerable attention because of the excellent permeability characteristics, far better than those of PET, i.e., furan-based polyesters, and in particular poly(ethylene furanoate) (PEF). Although known since the fifties, the great success of 2,5-furandicarboxylic acid (FDCA) is mainly due to the discovery of a green route for its production [4,5]. Today, furan-based monomers can be obtained from sugar dehydration in large amounts and high purity. The interest in these compounds is so high that the US Department of Energy included FDCA, 5-hydroxymethyl-furfural (HMF) and furfural in the top “10 + 4” list of bio-based chemicals [6]. The industry’s interest in these building blocks is high as well. Consequently, in fall 2016. Avantium and BASF announced the establishment of a joint venture called Synvina to build a plant with a capacity of 50,000 tons/year for the production of FDCA, the raw material for the synthesis of furanoate-based polymers [7].

The exceptional gas permeability behavior of PEF as compared to PET is linked to the different molecular structure and chain mobility. Indeed, it has been reported that the substitution of apolar benzene ring of PET with polar furan ring, together with nonlinear axis of ring rotation in PEF hinders the furan ring-flipping, thus preventing permeant diffusion [8].

Furthermore, recent studies have demonstrated the enzymatic degradability of PEF and PET, opening up new possibilities in eco-friendly industrial depolymerization processes for recycling purposes [9,10,11]. In particular, a recent work demonstrated the degradability of PEF by cutinase from *Humicola insolens*, leading to complete polymer solubilisation in 72 h of incubation [11].

In this contribution, we propose a novel homopolymer with high gas barrier properties as an *alter ego* of FDCA-based polyesters, i.e., poly(butylene 2,5-thiophenedicarboxylate) (PBTF). Both starting materials, 2,5-thiophenedicarboxylic acid (TFDCA) and 1,4-butanediol (BD), can be derived from renewable resources, thus obtaining a fully biobased polymer. TFDCA is industrially produced from the reaction of adipic acid with thionyl chloride [12,13]. In turn, adipic acid can be obtained from glucaric or muconic acid [14]. Lastly, bio-based BD is prepared by hydrogenation of succinic acid [15]. Molecular, thermal, structural, mechanical and barrier properties of PBTF have been deeply investigated and correlated with the chemical structure, in order to establish structure-property relationships. 

## 2. Materials and Methods

### 2.1. Materials

TFDCA (97%) was purchased from TCI (Tokyo, Japan). Hexafluoro-2-propanol, chloroform, methanol, BD (99%), titanium tetrabutoxide (TBT, 97%) and titanium isopropoxide (TTIP, 97%) were obtained from Sigma Aldrich (Saint Louis, MO, USA). TBT and TTIP were both distilled before use, while other products and used as received.

### 2.2. Polyester Synthesis

PBTF was prepared by two-step melt polycondensation by reacting TFDCA (15.2 g, 0.883 mol) with BD (19.9 g, 0.22 mol) in the presence of 200 ppm/g_polymer_ of TBT and 200 ppm/g_polymer_ of TTIP. A 200 mL glass reactor was placed in a silicon oil bath, and the reaction mixture was stirred at 100 rpm by a two-bladed centrifugal stirrer connected to a overhead motor (IKA-Werke GmbH & Co., Staufen, Germany). Nitrogen flow was applied and the temperature was set to 170 °C. When more than 90% of the water produced during esterification was distilled off (about 3 h), pressure and temperature were gradually reduced to 0.1 mbar and increased to 200 °C. Polymerization was stopped when constant torque was measured (4 additional hours).

As-synthesized polymer was purified through dissolution in a mixture hexafluoro-2-propanol/chloroform and precipitation in methanol. The purified polymer, in the form of white floccules, was dried at 30 °C under vacuum to constant weight. Thin films of about 150 μm thickness were obtained by compression molding using a Carver press. The purified polymer was melted at 180 °C and kept for 2 min at a pressure of 5 tons/m^2^. Lastly, the film was cooled to 23 °C in press by tap water.

Film thickness was determined by a Sample Thickness Tester DM-G (Brugger Feinmechanik GmbH, Munich, Germany). The reported value represents the mean thickness of three experimental tests, each run on 10 different points of the polymer film surface at RT.

### 2.3. Molecular, Thermal and Structural Characterization

Polymer structure was checked by ^1^H-NMR spectroscopy at RT. A Varian Inova (Palo Alto, CA, USA) 400-MHz was used for the measurements.

Molecular weight was determined by gel-permeation chromatography (GPC) at 30 °C with an Agilent 1100 HPLC system (Santa Clara, CA, USA) equipped with PLgel 5-μm MiniMIX-C column. A UV-detector was employed. A Hexafluoro-2-propanol/chloroform mixture (5:95 *v*/*v*) was used as eluent with a 0.3 mL/min flow. A molecular weight calibration curve was obtained with polystyrene standards in the molecular weight range 800–100,000 g/mol.

TGA was carried out under nitrogen atmosphere by means of a Perkin Elmer TGA7 apparatus (Waltham, MA, USA). Gas flow of 30 mL/min and heating scan of 10 °C/min were used for the analysis.

A Perkin Elmer DSC6 (Waltham, MA, USA) was used for the calorimetric measurements. Weighed samples were encapsulated in aluminum pans and heated to about 40 °C above fusion temperature at a rate of 20 °C/min (first scan), held there for 3 min, and then quenched to −40 °C. Finally, they were reheated from −10 °C to a temperature well above the melting at a heating rate of 20 °C/min (second scan). A heating rate of 60 °C/min was also used. Thermal annealing was performed at 65, 100, 130 and 145 °C and consisted of holding each sample for 10 min at the indicated temperature, followed by quenching and a scan collection (20 °C/min). Other specific thermal treatments are described in the text.

X-ray diffraction patterns of polymeric films were performed in the wide angle region by means of a PANalytical X’PertPro diffractometer equipped with a fast X’Celerator detector (Almelo, the Netherlands). The radiation was supplied by a copper target (λ = 0.1548 nm), and 567 points at interval 0.1° (2θ) were scanned for 100 s each. In situ XRD analysis was performed by using an Anton Paar TTK-450 (Graz, Austria) sample stage. Temperature was increased at 20 °C/min, and data collection was carried out as reported in the figures, by scanning from 10 to 35° 2θ degrees counting 40 s each 0.1° step (with the fast X’Celerator detector an XRD scan was collected in 40 s). The 1st temperature scan was performed from 23 °C up to 145 °C. After holding for 3 min at this temperature, the samples were quenched in liquid nitrogen. 

### 2.4. Mechanical Properties

Tensile measurements were carried out on rectangular films (5 mm wide and 0.2 mm thick) with a crosshead speed of 10 mm/min by using a Instron 4465 tensile testing machine (Norwood, MA, USA), equipped with a rubber grip and a 100 N load cell. A preload of 1 MPa was applied to each specimen prior to testing. At least five replicates were run, and the results are presented as the average ± standard deviation.

### 2.5. Color Evaluation

The color of film samples was measured using a HunterLab ColorFlex EZ 45/0° color spectro-photometer (Reston, VA, USA), with D65 illuminant, 10° observer (according to ASTM E308). Measurements were made using CIE Lab scale. The instrument was calibrated with a black and white tile before the measurements. Results are expressed as L* (lightness), a* (red/green) and b* (yellow/blue) parameters. The total color difference (ΔE) was calculated using the following equation:

ΔE = [(ΔL)^2^ + (Δa)^2^ + (Δb)^2^]^0.5^ (1)

where ΔL, Δa and Δb are the differentials between a sample color parameter (L*, a*, b*) and the color parameter of a standard white plate used as the film background (L′ = 66.39, a′ = −0.74, b′ = 1.25). Chromaticity C* = [(a*)^2^ + (b*)^2^]^0.5^ and hue angle *h*_ab_ = tan^−1^ (b*/a*) were determined. Measurements were carried out in triplicate at random positions over the film surface. 

### 2.6. Gas Permeability

Permeability tests were performed by the manometric method using a Permeance Testing Device, type GDP-C (Brugger Feinmechanik GmbH, München, Germany), in accordance with ASTM 1434-82, DIN 53 536 in compliance with ISO/DIS 15 105-1 and to Gas Permeability Testing Manual (Brugger Feinmechanik GmbH). After a preliminary high vacuum desorption of the system, the upper chamber was filled with the gas test at ambient pressure. A pressure transducer, set in the chamber below the film, recorded the increasing of gas pressure as a function of time. The gas transmission rate (GTR, expressed as cm^3^·cm·m^−2^·d^−1^·bar^−1^) was determined considering the pressure increase in relation to time and volume of the device.

All the measurements were carried out by using a gas stream of 100 cm^3^/min, 0% of gas RH. Food grade O_2_, CO_2_ and N_2_ were used. 

Permeability was determined at 5, 10, 15, 23, 35, 40, 45 and 50 °C. Experiments were performed at least in triplicate, and results are presented as the average ± standard deviation. Method A was used for the analysis, as reported in the literature [16,17]. Sample temperature was set by an external thermostat HAAKE-Circulator DC10-K15 type (Thermo Fisher Scientific, Waltham, MA, USA).

## 3. Results and Discussion

### 3.1. Molecular, Thermal and Structural Characterization

As synthesized, the polymer appeared as a yellowish hard solid material, while the purified one was obtained as white floccules. 

^1^H-NMR analysis confirmed the expected structure (Figure S1) and no impurities were detected in the spectrum. High molecular weight (*M*_n_ = 26,500 g/mol) and fairly low polydispersity (PDI = 2.5) were measured by GPC. This result confirms that optimized reaction conditions were achieved, especially when comparing PBTF molecular weight to that reported in the literature for PEF and PET synthesized by two-step melt polycondensation (*M*_n_ = 11,200 and 13,700 g/mol, respectivley) [18].

Therefore, melt polycondensation was revealed as a winning strategy for the preparation of highly pure PBTF. It is worth highlighting that the reagents were used as received and that the adopted protocol is very close to industrial procedures for the preparation of polyesters. In addition, by starting from dicarboxylic acid, the esterification of TFDCA and the consequent formation of methanol during polymerization were avoided. On the contrary, to obtain high molecular weight furanoate-based polymers, various authors reported the need for methyl-esterification of FDCA before polymerization [19,20,21].

PBTF displayed good thermal stability, higher than that of poly(butylene furanoate) (PBF) [22], up to 391 °C. At this temperature the degradation process started and reached its maximum rate at 411 °C. Degradation followed a one-step path (Figure S2) and no residual mass was detected at 750 °C. 

**Figure 1:**
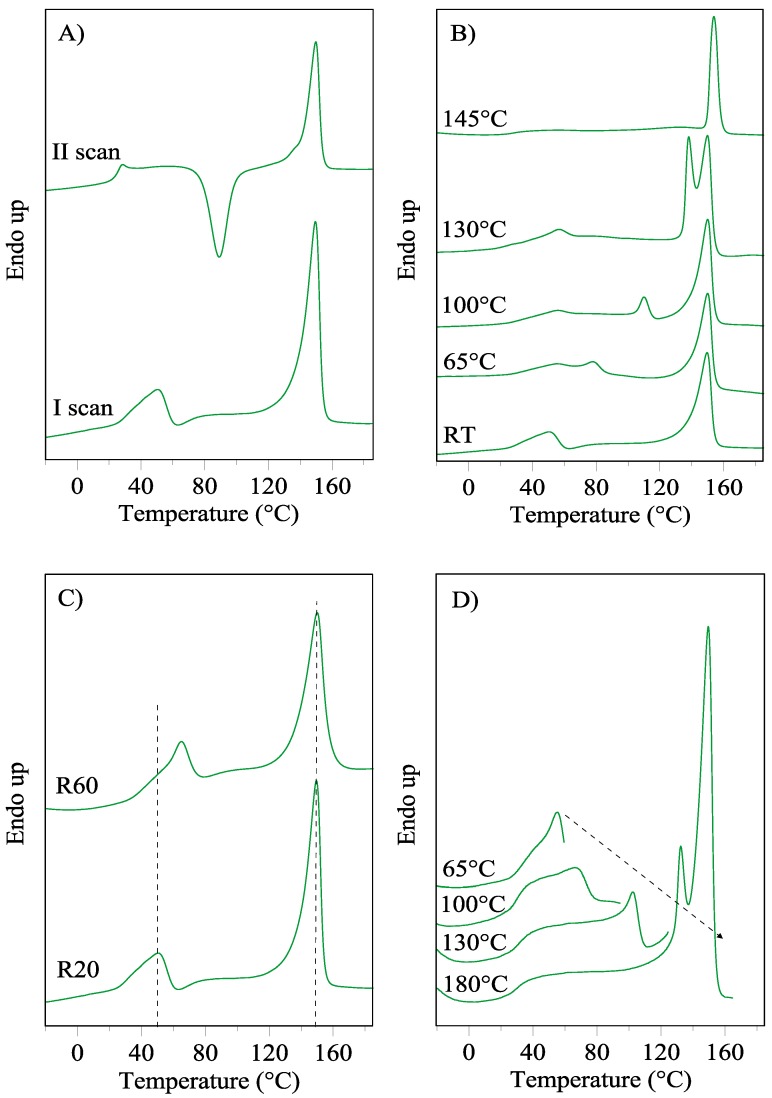
DSC curves of PBTF film: (**A**) I and II scan (20 °C/min); (**B**) untreated and annealed (10 min at the indicated temperatures) film; (**C**) different heating rates (20 and 60 °C/min, dotted lines represent eye guides); (**D**) consecutive heating steps to 65, 100, 130 and 180 °C.

The first DSC scan of the PBTF film provided evidence of the semicrystalline nature of this polyester (Figure 1A). The glass transition temperature is not clearly detectable, while an endothermic peak at about 50 °C (I) and a more pronounced one at higher temperature (II) have been highlighted (Table 1). The second DSC scan, recorded after melt quenching of the sample, is typical of an amorphous material that crystallizes during heating (Figure 1A). It presents the classic endothermic step ascribable to the glass transition located at 25 °C, an exothermic peak centered at 89 °C and an endothermic one located at 150 °C. 

The amorphous nature of the material after melt quenching is confirmed as the exothermic (Δ*H*_cc_) and endothermic (Δ*H*_m_) heats are both equal to 28 J/g (Table 1).

To shed light on the nature of the low temperature endothermic peak, annealing treatments were performed, and the calorimetric traces of the so-treated film samples are reported in Figure 1B.

After annealing, peak I shifted to higher temperature, while the position of peak II was not affected by the thermal treatment. The higher the annealing temperature, the sharper the peak I and the more consistent its temperature shift: from 51 to 78 °C, 109 and 140 °C, respectively, suggesting an improvement of its associated phase. Moreover, an intense baseline deviation due to the glass transition phenomenon became fully visible in the DSC scans of the annealed samples (Figure 1B), thus allowing for the detection of *T*_g_ (at 30 °C ca.).

In the case of the higher annealing temperature (145 °C), the calorimetric trace is characterized by the presence of only one endothermic phenomenon located at a higher temperature with respect to the most intense peak of the other annealed samples (peak II). This result could be explained as being due to: (i) an improvement of the phase corresponding to the endothermic peak located at 150 °C or to (ii) the development of a new ordered phase.

The effect of the heating rate on the two endothermic peaks was also evaluated. The corresponding calorimetric traces are reported in Figure 1C. A different behavior for the two endotherms can be highlighted: the position of peak I is dependent on the scanning rate, whereas that of peak II is not influenced. As is well known, the melting phenomenon is a first-order transition and, consequently, it does not depend on the heating rate. On the other hand, the dependence of a transition on the scanning rate is indicative of its second-order nature. Typical second-order transitions are the glass to rubber transitions and the melting of two-dimension ordered phases, like *meso*-phase. The presence of *meso*-phase has been already observed in other polyesters, such as PET [23,24] and polylactide (PLA) [25,26], although in these cases it mainly develops upon straining and/or thermal treatment.

To better understand the nature of the low temperature endothermic peak present in the DSC trace of PBTF film, the reversibility of this transition has been also verified. In this view, a PBTF film sample was subjected to the following thermal treatment (20 °C/min): heating step to 65, 100, and 130 °C, each followed by quenching to −40 °C and heating to the subsequent temperature of 100, 130, and 180 °C (Figure 1D). According to the intrinsic low crystallization rate of PBTF homopolymer (as clearly shown in Table 1 and Figure 1A, it can be frozen in its amorphous state by melt quenching), after the adopted thermal treatment, peak I should not appear if associated with a first-order transition. On the contrary, it is still evident in the subsequent DSC curve (Figure 1D). In particular, after heating to 100 °C (2nd cycle), peak I is visible, yet at a higher temperature. The same behavior was observed after the 3rd cycle (130 °C) and the 4th cycle (180 °C), with a continuous shift of peak I towards peak II. The observed behavior confirms the reversible character of the transition associated with peak I.

**Figure 2:**
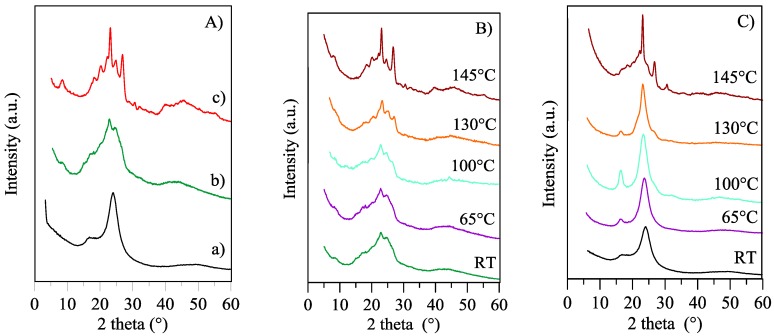
XRD patterns of PBTF: (**A**) from bottom to top, stretched film (a), film (b), well crystallized sample (c) obtained by slow cooling after melting; XRD scans performed at RT on untreated and annealed (10 min at the indicated temperatures) film (**B**) and stretched film (**C**).

X-ray diffraction (XRD) was carried out to clarify the nature of the ordered phases of PBTF. The XRD scanning at RT of PBTF film (Figure 2A-b) shows a low defined pattern: few, broad peaks of low intensity overlap on a bell-shaped intense background. The two most intense peaks are located at 2θ 22.9 and 24.7° (d = 3.88, 3.60 Å) and possible other peaks are located at 8.7, 15.1, 17.1° (d = 10.1, 5.8, 5.1 Å).

With the aim of improving the diffractometric pattern, PBTF film was subjected to tensile tests (this sample is hereafter defined as stretched film). Stretching is indeed an efficient tool to induce sample crystallization and, consequently, to improve the quality of the crystalline phase. After this treatment, XRD pattern of PBTF stretched film (Figure 2A-a) did not look like that of a tridimensional crystalline phase, as it displayed two partially overlapped very broad reflections, positioned at 16.7 and 24.2° (d = 5.3, 3.7 Å). On the contrary, such a profile is typical of a roughly 2D-ordered phase, hereinafter referred to as *meso*-PBTF. The comparison of film and stretched film diffraction profiles (Figure 2A) suggests the simultaneous presence of a crystalline phase and of a *meso*-phase in the film, this latter being the majority. Moreover, the elongation test performed on the film seems to favor the formation of the *meso*-PBTF phase over the crystalline one. 

**Figure 3:**
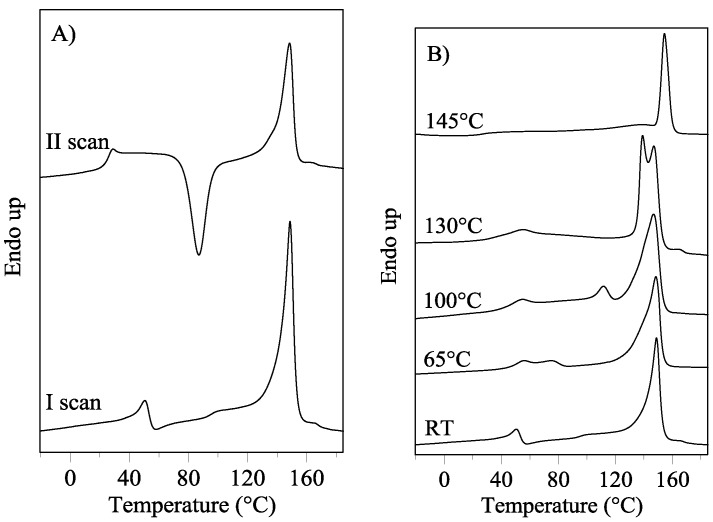

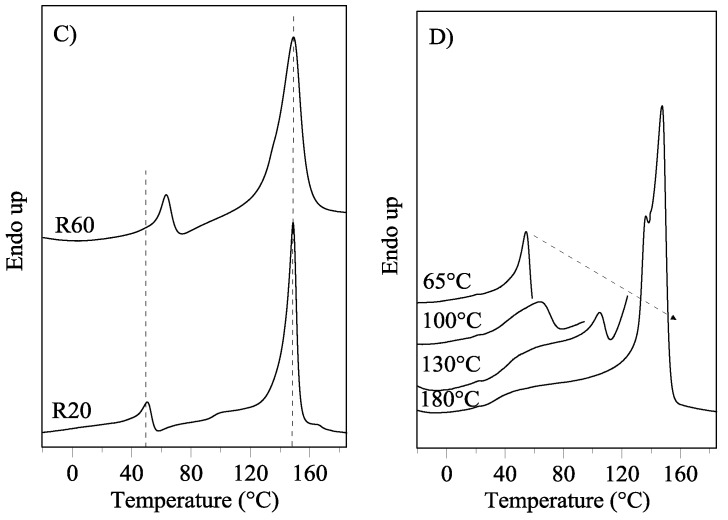
DSC curves of PBTF stretched film: (**A**) I and II scan (20 °C/min); (**B**) untreated and annealed (10 min at the indicated temperatures) stretched film; (**C**) different heating rates (20 and 60 °C/min, dotted lines represent eye guides); (**D**) consecutive heating steps to 65, 100, 130 and 180 °C.

The presence of *meso*-PBTF in the film is also supported by the calorimetric measurements carried out on the stretched film subjected to the same thermal treatments of the film. PBTF stretched film presented an analogous thermal behavior with respect to the film (Figure 3).

It is therefore possible to hypothesize that the reversible and second order low-temperature endothermic peak, characteristic of both PBTF film and stretched film, is related to the *meso*-phase evidenced by XRD analysis. To verify the above-mentioned statement, annealed PBTF films and stretched films were subjected to X-ray diffraction analysis.

The corresponding XRD patterns are reported in Figure 2B,C, respectively. XRD profiles of annealed film showed a progressive improvement of the ordered phase. In particular, the higher the annealing temperature, the higher the sharpness, intensity and definition of the peaks located at 23.1 and 26.9° (d = 3.84 and 3.31 Å). This effect is also accompanied by the increase of several additional reflections at 8.4, 18.2, 20.2, 22.2 and 24.8° (d = 10.5, 4.87, 4.39, 3.99, 3.60 Å) (Figure 2B).

The same trend characterizes the XRD profiles of annealed PBTF stretched films (Figure 2C). In this case, it is worth noting that the *meso*-phase underwent a continuous perfection as the temperature of the annealing treatment increased, the two peaks at 2θ 16.7 and 24.2° becoming sharper and more intense. Such improvement of the *meso*-phase occurred up to 100 °C. 

On the other hand, a decrease of the signal at 16.7°, associated with the *meso*-phase, together with the appearance of a shoulder at 26.6°, related to the crystal phase, was observed when an annealing temperature of 130 °C was used. Finally, stretched film annealed at 145° showed a profile typical of a semicrystalline polymer with the two main sharp peaks at 23.1 and 26.7°. These two peaks are also present in the XRD pattern of a well crystallized sample of PBTF, obtained by slow cooling after melting (Figure 2A-c), which is characterized by two major peaks at 23.1 and 26.8° (d = 3.84 and 3.32 Å) and several additional reflections at 8.4, 18.2, 20.2, 22.2 and 24.8° (d = 10.5, 4.87, 4.39, 3.99, 3.60 Å). Hereinafter, this phase is named α-PBTF.

**Figure 4:**
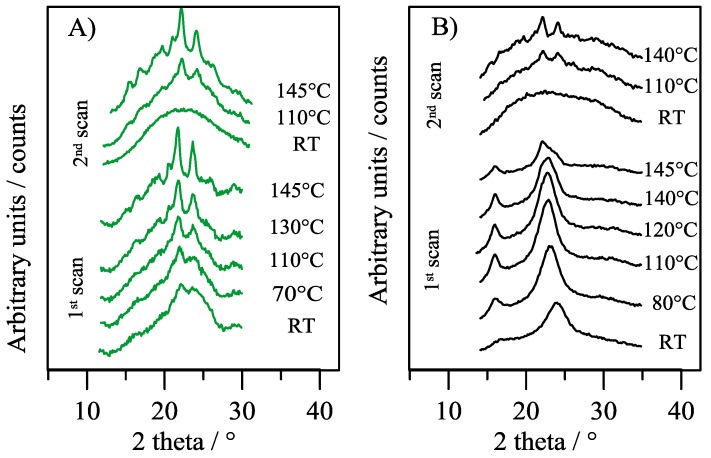
In situ XRD patterns collected at different temperatures (I scan and II scan after quenching from the melt) for PBTF film (**A**) and stretched film (**B**).

The stability of the crystalline phase of the film was investigated by in situ XRD temperature scans (Figure 4A).

In the first scan, a significant enhancement of peak intensities occurred as temperature increased, up to melting. Since the patterns show an evolution towards the full profile characteristic of the α crystal phase, it can be stated that the low intensity and broad reflections detected in the untreated film belong to this phase. After melt quenching, during the 2nd thermal scan, the sample, initially amorphous, crystallized arranging in the α-PBTF lattice. In situ XRD temperature scans were also carried out on PBTF stretched film. The results (Figure 4B) show that *meso*-phase peaks became sharper and more intense as the temperature increased, up to 100 °C, then at 140 °C a small worsening occurred. At 145 °C, a temperature very close to the melting point, significant changes of the in situ XRD pattern occurred, with a shift toward a profile reminiscent of a low crystalline α-PBTF.

The results of both the annealing treatment (Figure 2B) and of the in situ XRD temperature measurements (Figure 4A) of the film samples confirm that the *meso*-phase covers the largest fraction of the ordered phase. By proper thermal treatments, the fraction of the α-crystal phase can be increased, through the conversion of *meso*-phase into α-crystal phase, as already observed for poly(l-lactide) [27].

### 3.2. Mechanical Characterization and Color Evaluation

**Figure 5:**
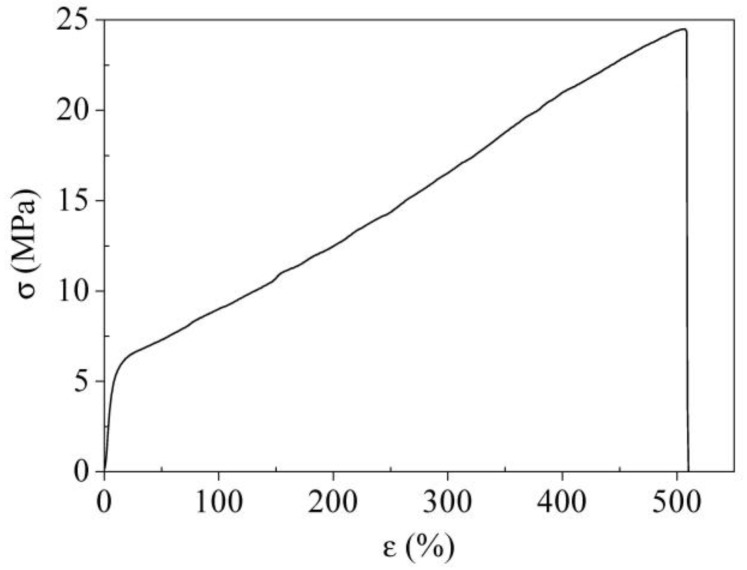
Representative stress-strain curve.

The mechanical behavior of PBTF, evaluated by tensile measurements on thin films, is presented in Figure 5. As can be seen, PBTF film displays high elongation (ε_b_ = 555% ± 50%) and stress at break (σ_b_ = 24.5 ± 0.5 MPa). In addition, no necking was recorded at the yield point, and the elastic modulus is equal to 89 ± 7 MPa. Thus, mechanical characteristics of PBTF are quite peculiar, especially as compared to that of its furanoate-based counterparts, i.e., PEF and PBF. PBTF exhibits a hard and tough behavior making it very interesting, e.g., for flexible packaging applications. On the contrary, PBF shows yield and high elastic modulus, above 800 MPa [22,28,29,30]. Similar characteristics, i.e., very high elastic modulus and brittle fracture, have been highlighted for PEF [19,31].

Such diverse properties for polymers with very similar chemical structure can be mainly ascribed to the different *T*_g_s and to the peculiar structural arrangement of PBTF polymer chains. Since tensile tests were carried out at room temperature, therefore below *T*_g_ for PBF (i.e., 35–40 °C) [30] and PEF (i.e., 77 °C) [19] and around *T*_g_ for PBTF, the polymers are respectively in the glassy (PBF and PEF) and in the rubbery state (PBTF). Moreover, as mentioned above, PBTF is characterized by the presence of the *meso*-phase that constitutes the majority of the ordered domains, thus lowering the overall degree of crystallinity.

Film transparency and color is very important, especially if food packaging application is envisioned. Given the high amount of pigments present in food, its color has been always considered one of the key factors used to evaluate quality and taste by the final consumer. Therefore, packaging should interfere as little as possible with the color of the product, not to cause a decrease in attractiveness. The results of the PBTF film surface color determination is reported in Table S1 as compared to a white standard. An explanation of the significance of the various parameters is also provided in SI. PBTF shows an L* close to white, while a* and b* indicate a faint tendency toward a yellowish color (*h*_ab_ over 90°), as due to the presence of sulphur atoms along the polymer backbone. A very low C* has been recorded, meaning low color saturation and thus a good transparency of the film.

### 3.3. Gas Permeability

As reported in the literature [32], several factors affect film gas permeability, such as molecular structure, chain stiffness, molecular symmetry, degree of order, crystallinity and orientation, presence of cross-linking, glass transition temperature. It is well known that gas transmission through a polymeric material is linked to the tortuosity induced by impermeable domains, like the crystalline phase, within the polymer matrix.

**Figure 6:**
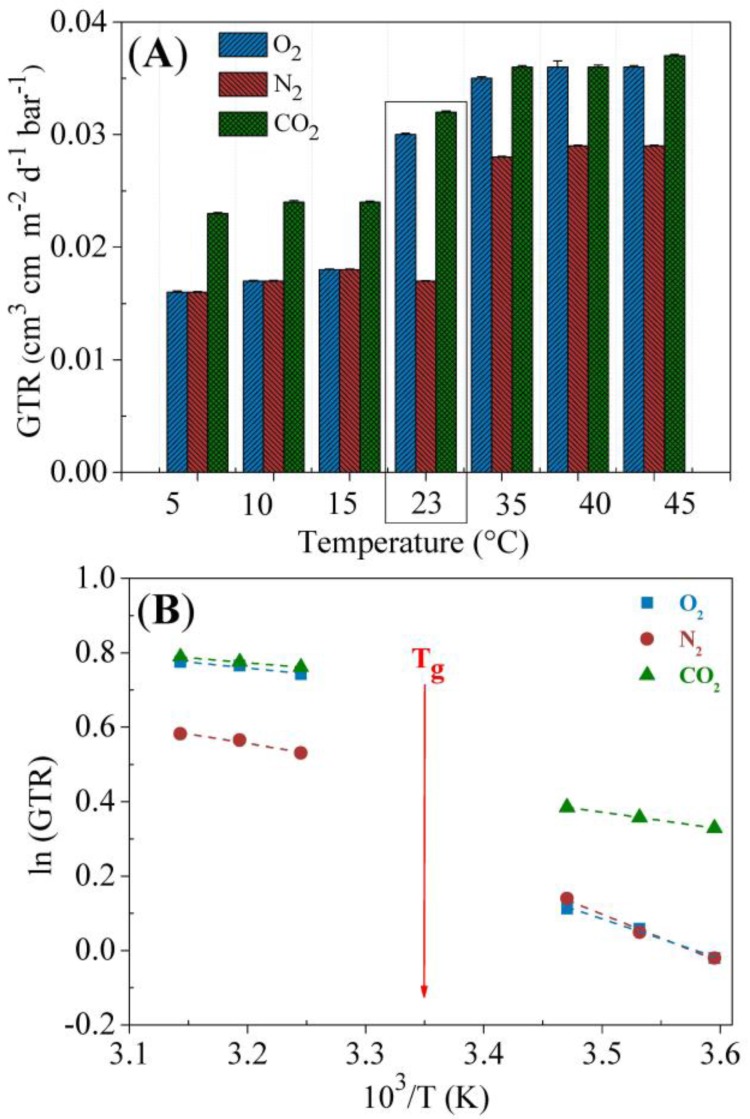
Gas transmission rate (**A**) and Arrhenius plot of GTR (**B**) in the range 5–45 °C for CO_2_, O_2_, and N_2_.

To evaluate the diffusion of small molecules through PBTF film, barrier properties have been investigated using N_2_, O_2_ and CO_2_ gas, due to their different van der Waals molar volume and inertness towards organic polymers. The collected data are reported in Figure 6A and Table S2 as gas transmission rate (GTR).

As expected, GTR depends on the temperature and, more specifically, it increases by enhancing the temperature. In particular, given the near-ambient temperature *T*_g_ of PBTF (i.e., 25 °C), two different behaviors can be outlined: below *T*_g_ and above *T*_g_. In addition, although very close to *T*_g_, GTR was also measured at 23 °C since it is a very common temperature used for barrier properties evaluation.

A sudden increase in permeability was recorded at this temperature, while a lower dependence on T can be observed both in the range 5–15 °C and 35–45 °C. 

Below *T*_g_, the chain segments have little mobility; therefore, gas molecules must follow a more tortuous path to diffuse through the polymer matrix, also because of the reduction in free volume. Thus, GTR in the range 5–15 °C is lower than at 23 °C or above.

It is worth noticing that the barrier performances of PBTF remain very high in all the explored ranges of temperature, both below and above *T*_g_, thus broadening its range of possible operating conditions. This result can be ascribed to the presence, together with the crystalline domains, of the *meso*-phase that acts as an additional obstacle for the diffusion of the gas molecules, because of the dense packing of the macromolecular chains in this 2D-ordered phase.

Perm-selectivity ratios (Table S2) are not constant throughout all the temperature range and rather show a certain dependence on the measuring temperature, confirming that this parameter is correlated not only with the chemical structure of the material, but also with *T* [33].

When compared to the barrier properties of other polyesters (Table S3), the values found for PBTF acquire much more value. Indeed, O_2_ and CO_2_ permeabilities are well below those of PLA, PEF and amorphous PET and comparable to those already excellent of PPF [34,35,36,37]. 

For temperatures where no transitions in polymers (e.g., glass transition) and in permeants (e.g., boiling point) are detected, the permeation dependence on the temperature can be described through the Arrhenius model [34]. A linear correlation between the logarithm of a transport parameter and the reciprocal of the absolute temperature exists:

*P* = *P*_0_exp (−*E*_p_/*RT*) (2)

where *P* is the gas permeability (GTR), *P*_0_ is a pre-exponential factor of permeation, *E*_p_ is the activation energy for permeation and *R* is the gas constant.

As mentioned above, gas permeability changes across polymer *T*_g_. *E*_p_ can therefore be greater or smaller above *T*_g_ than below *T*_g_ [38].

*E*_p_ was then calculated in the two following ranges of temperature: 5–15 °C (below *T*_g_) and 35–45 °C (above *T*_g_). Figure 6B reports the GTR dependence of the studied gases on the temperature according to Equation (2). From the linear fitting of the experimental data (dashed lines) the activation energies have been calculated and reported in Table 2. Experimental data well fit the theoretical behavior, thus indicating a good correlation between permeability and temperature for all gases.

*E*_p_ values vary for all gases by changing the temperature range from below to above *T*_g_. However, different trends can be highlighted. *E*_p_ for O_2_ and N_2_ follows a similar path: a higher *E*_p_ is observed below *T*_g_, while in the range 35–45 °C a lower dependence of GTR on the temperature has been detected (lower values of E_p_). On the contrary, for CO_2_, *E*_p_ remains similar both in the range above *T*_g_ and below *T*_g_.

However, in both temperature ranges, a clear dependence on the permeant size can be observed, as *E*_p_ decreases with the increase of the permeant size in the following order: CO_2_ < O_2_ < N_2_, as already observed in the literature [39]. Furthermore, above *T*_g_, the activation energies of the permeation process for the three gases are less influenced by the permeant size, probably because of the enhanced mobility of the macromolecular chains that allows for an easier crossing of the gas molecules.

Although further investigations are necessary for a comprehensive characterization of the gas permeability behavior, the results presented provide meaningful evidence for the potential of PBTF as a high gas barrier polymeric film.

## 4. Conclusions

Poly(butylene 2,5-thiophene dicarboxylate), an aromatic polyester derived from renewable resources, has been successfully synthesized through melt polycondensation. PBTF shows good thermal properties, i.e., stability above 390 °C, glass transition at around room temperature (25 °C) and melting point of 150 °C, coupled with an intriguing mechanical behavior. The absence of yielding point, elongation at break above 500% and stress at break of 25 MPa, together with an elastic modulus of about 90 MPa, have been indeed demonstrated by tensile testing.

In addition, with respect to PEF, PBTF displays a reduction of permeability to O_2_ and CO_2_ of 2.0× and 4.75×, respectively [34,35].

These exceptional barrier properties are maintained both above and below *T*_g_, owing to the peculiar structural arrangement that allows for the formation of a not-induced 2D-ordered phase, i.e., *meso*-phase, and of 3D-crystalline domains, named α-PBTF. The former, together with the effect provided by the crystalline regions, causes a dense packing of PBTF macromolecules, thus hampering the gas permeation. 

Calorimetric and diffractometric studies evidenced that the *meso*-phase is highly stable, up to 140 °C, although by annealing it is possible to increase the amount of α-phase, by inducing a conversion of the *meso*-phase into α-PBTF. Furthermore, the α-crystal phase is the most thermodynamically stable since, after melt quenching, it is the only one that forms.

Thanks to the combination of all the above-mentioned characteristics, PBTF establish itself as a very important member of the biobased polyester family, opening up new possibilities in sustainable packaging, particularly for flexible films purposes.

## Figures and Tables

**Table 1:** I and II calorimetric scan data of PBTF film and stretched film.

	I Scan				II Scan					
Sample	*T*_m,I_ (°C)	Δ*H*_m,I_ (J/g)	*T*_m,II_ (°C)	Δ*H*_m,II_ (J/g)	*T*_g_ (°C)	Δ_Cp_ (J/g°C)	*T*_cc_ (°C)	Δ*H*_cc_ (J/g)	*T*_m_ (°C)	Δ*H*_m_ (J/g)
film	51	4	150	30	25	0.287	89	28	150	28
stretched film	51	4	149	45	25	0.283	87	25	150	25

**Table 2:** Activation energy of the gas transmission rate process for O_2_, N_2_ and CO_2_. In brackets: *R*^2^ coefficient.

Temperature range (°C)	*E*_p_ (kJ/mol)		
	O_2_	N_2_	CO_2_
5–15	8.91 (0.98)	10.65 (0.98)	3.73 (0.99)
35–45	2.68 (0.89)	4.21 (0.93)	2.25 (0.99)

## Supplementary Materials

The following are available online at http://www.mdpi.com/2073-4360/10/2/167/s1, Figure S1: 1H-NMR spectrum of PBTF with resonance assignments, Figure S2. Thermogravimetric curve (solid line) under nitrogen flow of PBTF and its derivative (dashed line), Table S1: L*, a*, b*, total color difference (ΔE), C* and hab of PBFT film (film color determination data), Table S2: Gas transmission rate (GTR) of N_2_, O_2_ and CO_2_ in the range 5–45 °C and perm-selectivity ratios for PBTF film, Table S3: Gas transmission rate (GTR) of PBTF with respect to other polyesters.

## References

[1] (2016). PlasticsEurope—Plastics the Fact, Brussels, Belgium. www.plasticseurope.org.

[2] Siracusa V., Rocculi P., Romani S., Dalla Rosa M. (2008). Biodegradable polymers for food packaging: A review. Trends Food Sci. Technol..

[3] European Bioplastics—Bioplastics Facts and Figures, Berlin, Germany. http://en.european-bioplastics.org.

[4] Gandini A., Silvestre A.J.D., Neto C.P., Sousa A.F., Gomes M. (2009). The furan counterpart of poly(ethylene terephthalate): An alternative material based on renewable resources. J. Polym. Sci. Pol. Chem..

[5] Papageorgiou G.Z., Papageorgiou D.G., Terzopoulou Z., Bikiaris D.N. (2016). Production of bio-based 2,5-furan dicarboxylate polyesters: Recent progress and critical aspects in their synthesis and thermal properties. Eur. Polym. J..

[6] Van Putten R.J., Van der Waal J.C., De Jong E., Rasrendra C.B., Heeres H.J., De Vries J.G. (2013). Hydroxymethylfurfural, A Versatile Platform Chemical Made from Renewable Resources. Chem. Rev..

[7] Synvina: Joint Venture of BASF and Avantium Established. www.basf.com.

[8] Burgess S.K., Leisen J.E., Kraftschik B.E., Mubarak C.R., Kriegel R.M., Koros W.J. (2014). Chain mobility, thermal, and mechanical properties of poly(ethylene furanoate) compared to poly(ethylene terephthalate). Macromolecules.

[9] Ferrario V., Pellis A., Cespugli M., Guebitz G.M., Gardossi L. (2017). Nature Inspired Solutions for Polymers: Will Cutinase Enzymes Make Polyesters and Polyamides Greener?. Catalysts.

[10] Pellis A., Gamerith C., Ghazaryan G., Aortner A., Herrero Acero E., Guebitz G.M. (2016). Ultrasound-enhanced enzymatic hydrolysis of poly(ethylene terephthalate). Bioresour. Technol..

[11] Weinberger S., Canadell J., Quartinello F., Yeniad B., Arias A., Pellis A., Guebitz G.M. (2017). Enzymatic Degradation of Poly(ethylene 2,5-furanoate) Powders and Amorphous Films. Catalysts.

[12] Zhi W., Hu Y., Liang M., Liu Y., Li J., Yin J., Shi Y. (2014). Solid-liquid equilibrium and thermodynamic of 2,5-thiophenedicarboxylic acid in different organic solvents. Fluid Phase Equilibria.

[13] Yang Y., Zhang Q., Cao C., Cheng L., Shi Y., Yang W., Hu Y. (2014). Solubility and solution thermodynamics of 2,5-thiophenedicarboxylic acid in (water + ethanol) binary solvent mixtures. Thermochim. Acta.

[14] Polen T., Spelberg M., Bott M. (2013). Toward biotechnological production of adipic acid and precursors from biorenewables. J. Biotechnol..

[15] Choi S., Song C.W., Shin J.H., Lee S.Y. (2015). Biorefineries for the production of top building block chemicals and their derivatives. Metab. Eng..

[16] Siracusa V. (2012). Food packaging permeability behaviour: A report. Int. J. Polym. Sci..

[17] (2008). Gas Permeability Testing Manual.

[18] Papageorgiou G.Z., Tsanaktsis V., Bikiaris D.N. (2014). Synthesis of poly(ethylene furandicarboxylate) polyester using monomers derived from renewable resources: Thermal behavior comparison with PET and PEN. Phys. Chem. Chem. Phys..

[19] Knoop R.J.I., Vogelzang W., Van Haveren J., Van Es D.S. (2013). High molecular weight poly(ethylene-2,5-furanoate); Critical aspects in synthesis and mechanical property determination. J. Polym. Sci. Pol. Chem..

[20] Papageorgiou G.Z., Tsanaktsis V., Papageorgiou D.G., Exarhopoulos S., Papageorgiou M., Bikiaris D.N. (2014). Evaluation of polyesters from renewable resources as alternatives to the current fossil-based polymers. Phase transitions of poly(butylene 2,5-furan-dicarboxylate). Polymer.

[21] Tsanaktsis V., Papageorgiou G.Z., Bikiaris D.N. (2015). A facile method to synthesize high-molecular-weight biobased polyesters from 2,5-furandicarboxylic acid and long-chain diols. J. Polym. Sci. Pol. Chem..

[22] Soccio M., Costa M., Lotti N., Gazzano M., Siracusa V., Salatelli E., Manaresi P., Munari A. (2016). Novel fully biobased poly(butylene 2,5-furanoate/diglycolate) copolymers containing ether linkages: Structure-property relationships. Eur. Polym. J..

[23] Ran S., Wang Z., Burger C., Chu B., Hsiao B.S. (2002). Mesophase as the precursor for strain-induced crystallization in amorphous poly(ethylene terephthalate) film. Macromolecules.

[24] Keum J.K., Kim J., Lee S.M., Song H.H., Son Y.K., Choi J.I., Im S.S. (2003). Crystallization and transient mesophase structure in cold-drawn PET stretched films. Macromolecules.

[25] Stoclet G., Seguela R., Lefebvre J.M., Rochas C. (2010). New insights on the strain-induced mesophase of Poly(d,l-lactide): In situ WAXS and DSC study of the thermo-mechanical stability. Macromolecules.

[26] Lv R., Na B., Tian N., Zou S., Li Z., Jiang S. (2011). Mesophase formation and its thermal transition in the stretched glassy polylactide revealed by infrared spectroscopy. Polymer.

[27] Lv R., Zou S., Na B., Deng H., Yu Z. (2013). Influence of mesophase–crystal transition on thermal and mechanical properties of stretched Poly(l-lactide). Polym. Eng. Sci..

[28] Zhou W., Zhang Y., Xu Y., Wang P., Gao L., Zhang W., Ji J. (2014). Synthesis and characterization of bio-based poly(butylene furandicarboxylate)-b-poly(tetramethylene glycol) copolymers. Polym. Degrad. Stab..

[29] Wu L., Mincheva R., Xu Y., Raquez J.M., Dubois P. (2012). High molecular weight Poly(butylene succinate-*co*-butylene furandicarboxylate) copolyesters: From catalyzed polycondensation reaction to thermomechanical properties. Biomacromolecules.

[30] Zhu J., Cai J., Xie W., Chen P.H., Gazzano M., Scandola M., Gross R.A. (2013). Poly(butylene 2,5-furan dicarboxylate), a biobased alternative to PBT: Synthesis, physical properties, and crystal structure. Macromolecules.

[31] Jiang M., Liu Q., Zhang Q., Ye C., Zhou G. (2012). A series of furan-aromatic polyesters synthesized via direct esterification method based on renewable resources. J. Polym. Sci. Pol. Chem..

[32] Robertson G.L. (2006). Food Packaging: Principles and Practice.

[33] Siracusa V., Ingrao C. (2017). Correlation amongst gas barrier behaviour, temperature and thickness in BOPP films for food packaging usage: A lab-scale testing experience. Polym. Test..

[34] Burgess S.K., Karvan O., Johnson J.R., Kriegel R.M., Koros W.J. (2014). Oxygen sorption and transport in amorphous poly(ethylene furanoate). Polymer.

[35] Burgess S.K., Kriegel R.M., Koros W.J. (2015). Carbon dioxide sorption and transport in amorphous Poly(ethylene furanoate). Macromolecules.

[36] Vannini M., Marchese P., Celli A., Lorenzetti C. (2015). Fully biobased poly(propylene 2,5-furandicarboxylate) for packaging applications: Excellent barrier properties as a function of crystallinity. Green Chem..

[37] Siracusa V., Blanco I., Romani S., Tylewicz U., Rocculi P., Dalla Rosa M. (2012). Poly(lactic acid)-modified films for food packaging application: Physical, mechanical, and barrier behaviour. J. Appl. Polym. Sci..

[38] Komatsuka T., Nagai K. (2009). Temperature dependence on gas permeability and permselectivity of poly(lactic acid) blend membranes. Polym. J..

[39] Fu S., Sanders E.S., Kulkami S.S., Wenz G.B., Koros W.J. (2015). Temperature dependence of gas transport and sorption in carbon molecular sieve membranes derived from four 6FDA based polyimides: Entropic selectivity evaluation. Carbon.

