# Supplementary material for: Poly(butylene 2,5-thiophenedicarboxylate): An Added Value to the Class of High Gas Barrier Biopolyesters
Source: Polymers (Basel). 2018 Feb 9;10(2):167. doi: 10.3390/polym10020167 (PMC6414998; doi:10.3390/polym10020167)
Supplement: Supplementary file 1 [file polymers-10-00167-s001.pdf]

polymers

Supporting Information For

Poly(butylene 2,5-thiophenedicarboxylate): an Added Value to the Class of High Gas Barrier Biopolyesters

Giulia Guidotti [1], Matteo Gigli [2,*], Michelina Soccio [1], Nadia Lotti [1,*], Massimo Gazzano [3], Valentina Siracusa [4] and Andrea Munari [1]

1 Department of Civil, Chemical, Environmental and Materials Engineering, University of Bologna, Via Terracini 28, 40131 Bologna, Italy; giulia.guidotti9@unibo.it (G.G.); m.soccio@unibo.it (M.S.); andrea.munari@unibo.it (A.M.)
2 Department of Chemical Science and Technologies, University of Rome Tor Vergata, Via della Ricerca Scientifica 1, 00133 Roma, Italy;
3 Organic Synthesis and Photoreactivity Institute, CNR, Via Gobetti 101, 40129 Bologna, Italy; massimo.gazzano@cnr.it
4 Department of Chemical Science, University of Catania, Viale A. Doria 6, 95125 Catania, Italy; vsiracus@dmfci.unict.it
* Correspondence: matteo.gigli@uniroma2.it (M.G.); nadia.lotti@unibo.it (N.L.); Tel.: +39-067-259-4488 (M.G.); +39-051-209-0354 (N.L.)

Film Color Determination

In the Lab Color scale, the lightness coefficient (L*) ranges from black (0) to white (100). For any L* value, the coordinates a* and b* situate the color on a rectangular coordinate grid perpendicular to L* axis. At the origin (a* = 0 and b* = 0) the color is achromatic (gray). Moving on the horizontal axis, a positive a* value indicates a hue of red-purple, a negative a* value indicates a hue of green. Moving on the vertical axis, a positive b* value indicates a hue of yellow, a negative b* value indicates a hue of blue (McGuire, R.G. Reporting of objective color measurements. HortScience 1992, 27, 1254-1255, 10.1016/S0190-9622(89)80314-7).

Table S1. L*, a*, b*, total color difference (ΔE), C* and h_{ab} of PBFT film.

Sample	L*	a*	b*	ΔE	C*	h_{ab}
**white standard**	66.4	-0.74	1.25	-	1.45	121
**PBTF**	62.5 ± 0.3	-0.47 ± 0.02	6.46 ± 0.37	6.53	6.48	94

h_{ab} = 0°, red-purple; h_{ab} = 90°, yellow; h_{ab} = 180°, green; h_{ab} = 270°, blue.

Table S2. Gas transmission rate (GTR) of N_2, O_2 and CO_2 in the range 5–45°C and perm-selectivity ratios for PBTF film (film thickness of 166 μm).

**Temperature (°C)**	**GTR (cm³·cm·m⁻²·d⁻¹·bar⁻¹)**			**Perm-selectivity**		
	**O_2**	**N_2**	**CO_2**	**CO_2/O_2**	**CO_2/N_2**	**N_2/O_2**
**5**	0.016 ± 13E-5	0.016 ± 8E-5	0.023 ± 8E-5	1.4	1.4	1.0
**10**	0.017 ± 8E-5	0.017 ± 8E-5	0.024 ± 13E-5	1.4	1.4	1.0
**15**	0.018 ± 2E-5	0.018 ± 2E-5	0.024 ± 3E-5	1.3	1.3	1.0
**23**	0.025 ± 10E-5	0.023 ± 3E-5	0.032 ± 10E-5	1.3	1.4	1.0
**35**	0.035 ± 13E-5	0.028 ± 7E-5	0.036 ± 13E-5	1.0	1.3	0.8
**40**	0.036 ± 55E-5	0.029 ± 7E-5	0.036 ± 18E-5	1.0	1.2	0.8
**45**	0.036 ± 13E-5	0.029 ± 7E-5	0.037 ± 13E-5	1.0	1.3	0.8

Table S3. Gas transmission rate (GTR) of PBTF with respect to other polyesters.

**Polymer**	**O_2 GTR, 35 °C (cm³·cm·m⁻²·d⁻¹·bar⁻¹)**	**CO_2 GTR, 35 °C (cm³·cm·m⁻²·d⁻¹·bar⁻¹)**
**PBTF**	0.035[a]	0.036[a]
**PEF**	0.0702[b]	0.171[c]
**PPF**	< 0.005 – 0.0472[d]	/
**PET**	0.749[b]	3.22[c]
**PLA**	1.32[e]	3.24[e]

[a]this work; [b][32]; [c][33]; [d][34], values dependent on the ageing time, RH50; [e][35], 23 °C.

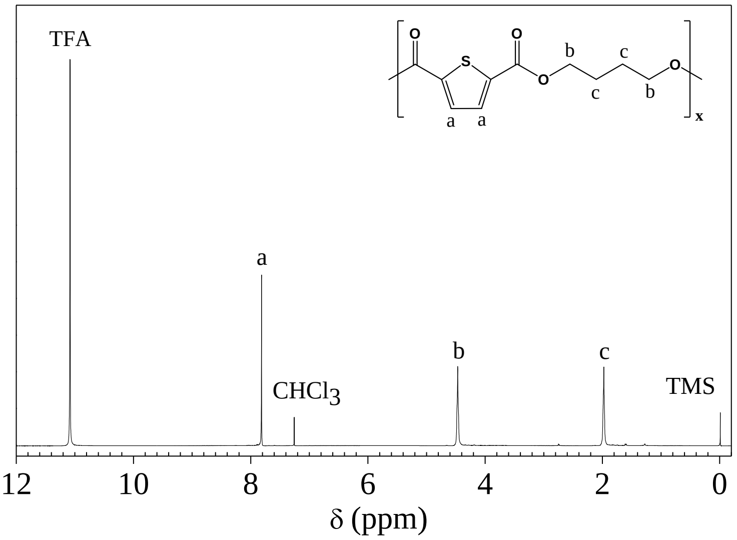

Figure S1. ¹H-NMR spectrum of PBTF with resonance assignments.

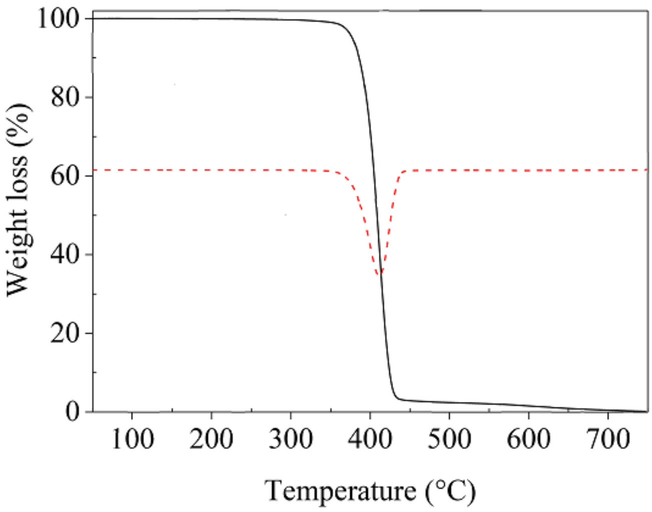

Figure S2. Thermogravimetric curve (solid line) under nitrogen flow of PBTF and its derivative (dashed line).
